# Investigation of the Effect of the Skull in Transcranial Photoacoustic Imaging: A Preliminary Ex Vivo Study

**DOI:** 10.3390/s20154189

**Published:** 2020-07-28

**Authors:** Rayyan Manwar, Karl Kratkiewicz, Kamran Avanaki

**Affiliations:** 1 Richard and Loan Hill Department of Bioengineering, University of Illinois at Chicago, Chicago, IL 60607, USA; r.manwar@wayne.edu; 2 Department of Biomedical Engineering, Wayne State University, Detroit, MI 48201, USA; karl.kratkiewicz@wayne.edu; 3 Department of Dermatology, University of Illinois at Chicago, Chicago, IL 60607, USA

**Keywords:** transcranial, skull bone, aberration, photoacoustic, distortion, brain imaging

## Abstract

Although transcranial photoacoustic imaging (TCPAI) has been used in small animal brain imaging, in animals with thicker skull bones or in humans both light illumination and ultrasound propagation paths are affected. Hence, the PA image is largely degraded and in some cases completely distorted. This study aims to investigate and determine the maximum thickness of the skull through which photoacoustic imaging is feasible in terms of retaining the imaging target structure without incorporating any post processing. We identify the effect of the skull on both the illumination path and acoustic propagation path separately and combined. In the experimental phase, the distorting effect of ex vivo sheep skull bones with thicknesses in the range of 0.7~1.3 mm are explored. We believe that the findings in this study facilitate the clinical translation of TCPAI.

## 1. Introduction

Transcranial imaging is considered as a significant milestone in the understanding of the underlying brain functionality. Transcranial Ultrasonography (TCUS) is a clinically approved non-invasive and rapid technique for the real-time measurement of cerebral blood flow characteristics in neonates [1,2,3]. TCUS is effective due to the very thin skull thickness in neonates. TCUS is the preferred modality to image the neonatal brain due to its portability, low cost, speed, and lack of ionizing radiation [4]. TCUS operates in low frequencies (0.5–2 MHz) to have sufficient skull penetration [5]. Among the pre-existing potential alternatives, intraoperative x-ray or CT may be used to navigate through bony anatomy [1,6,7]. Intraoperative magnetic resonance imaging (MRI) is another costly option [7]. X-ray, CT, and MRI all require sedation and exposure to ionizing radiation [6].

Photoacoustic imaging (PAI) has proved to be a promising tool for the diagnosis, prognosis, and treatment monitoring of neurological disorders in small and large animals [8,9,10,11,12,13,14]. PAI is a non-ionizing hybrid imaging modality based on the photoacoustic (PA) effect. PAI combines the high absorption contrast of optical imaging with the high spatial resolution of ultrasound imaging to visualize tissue chromophores in the optical quasi-diffusive or diffusive regime [15,16]. In PAI, the biological tissue is illuminated with a short-pulsed laser beam, generating acoustic waves via transient thermoelastic expansion [9,17,18,19]. The subsequent ultrasound waves propagating from within the tissue are then detected by an ultrasonic transducer array located outside the tissue. The ultrasound signals are used to form an image through a reconstruction algorithm [20]. Generated acoustic waves travel through the skull one way, unlike pulse-echo ultrasound. As a result, the waves are less susceptible to the attenuation that occurs when they encounter the skull–tissue interface [21,22].

One of the obstacles for PAI in transcranial imaging is the presence of the skull bone [23,24]. Skull bone represents a highly acoustical impedance mismatch and dispersive barrier for the propagation of acoustic waves [25]. The skull distorts the amplitude and phase of the received acoustic waves [26]. This distortion is contributed by four different phenomena: (i) the acoustic attenuation (i.e., the decrease in the acoustic signal amplitude) due to the absorption and scattering of the skull tissue [27,28,29]; (ii) the acoustic dispersion (i.e., the dependency of the speed of sound on frequency) modifies the phase of the acoustic wave [29]; (iii) the signal broadening, which is a frequency-dependent reduction in the acoustic wave amplitude [30]; and (iv) the temporal shift, where the significantly higher speed of sound in the bone (~2900 m/s [31]) as compared to the brain’s soft tissue (~1500 m/s [32]) makes the acoustic waves travel faster through the skull and be detected earlier. The degree of attenuation, dispersion, broadening, and temporal shift are determined by the mechanical properties of the skull (i.e., bone type, density, porosity, and thickness), among which the tissue thickness has the most significant effect [33,34,35]. In transcranial photoacoustic imaging, there are two sources of signal attenuation: (1) acoustic, and (2) optical. Acoustic attenuation can be represented by $A=A_{0}e^{-\alpha d}$, where $A_{0}$ is the signal amplitude before attenuation, d is the depth or thickness, and α is the attenuation coefficient. The attenuation coefficient is a function of frequency and is defined as: $\alpha =\omega^{2}\eta /2c_{p}$, where ω is the angular frequency, η is the viscosity, and $c_{p}$ is the phase velocity. Therefore, if the frequency or depth or both increase, the attenuation increases. Optical attenuation is studied based on the absorbing and scattering effects of the skull. The absorbing effect of the skull tissue can be represented by $A_{abs}=\varepsilon lC$, where ε is the molar absorptivity, l is the optical path length, and C is the concentration of the medium. The scattering effect of the tissue is a more complex event, and is modelled using the Extended Huygens–Fresnel (EHF) principle [36]. Studies have shown that the primary effect of scattering is a less steep slope of light intensity decay with depth than that predicted by the so-called single-scattering model that follows an exponential decay trend. A higher density causes increased optical and acoustic absorption, whereas a higher porosity causes more scattering [37].

The angle between the incident acoustic wave and the skull tissue affects the PA intensity. With increasing the incident angle, more shear waves are generated as compared to longitudinal waves, and hence the amplitude of the PA signal drops further. Yang and Wang et al. [38], evaluated the PA signal amplitude at two different frequencies (i.e., 1 and 2.25 MHz) as a function of the incident angle on the monkey’s skull, and found that increasing the incident angle up to ~35° decreases the PA signal, whereas beyond that angle the PA signal amplitude starts increasing again. This phenomenon is applicable if the boundaries are part of a layered material (such as skull tissue), where the longitudinal waves first convert into shear waves at the tissue–skull interface and later the shear waves convert back to longitudinal waves (mode conversion) at the skull–tissue interface and vice-versa.

Due to the distorting effects of the skull, the PAI of a small animal brain (with semi optically and acoustically transparent skull) has been conducted [39]; however, there are only a few studies to validate the feasibility of photoacoustic technology for transcranial imaging in animals with thicker skulls [40,41]. Several PA signal/image enhancement algorithms were developed to improve the quality of the degraded images due to the presence of the skull [42]. Although some of the algorithms were effective, they were computationally expensive and were not run in real-time. Therefore, determining the maximum skull thickness that would allow the imaging target to be accurately reconstructed without any post-processing is essential.

**Figure 1:**
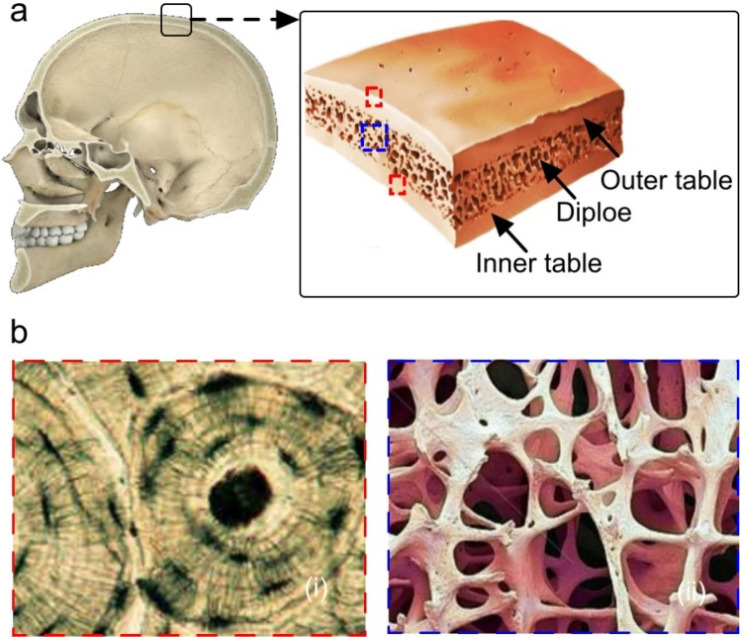
Skull bone structure. (**a**) Structural component of human skull bone, each layer magnified in (**b**i) the outer tables and (**b**ii) diploe.

In order to characterize the effect of the skull on the PAI, first we describe the skull bone structure and the corresponding physio-mechanical properties. The skull bone consists of three layers: the inner table, the middle diploe, and the outer table (see Figure 1a). The inner and outer table are cortical bones, whereas the middle diploe layer is the trabecular bone type [43]. The cortical and trabecular bones are anatomically different. At birth, the bones of the cranial vault are unilaminar tables (cortical type) and, thereafter, the intervening diploe (trabecular type) appears at about the fourth year [44]. With the age, the trabecular layer grows at a faster rate as compared to the cortical layers (26% volume per year turnover rate for trabecular and 3% for cortical bone) [45]. Cortical bone is a fairly solid (Figure 1b(i)) and dense material which consists of a minerals, organic parts, and water. The mineral ingredient is hydroxyapatite, and the organic parts are fibrous protein collagen and non-collagenous [46,47]. Trabecular bone primarily consists of lamellar, which are arranged in packets that make up an interconnected irregular array of plates and rods called trabeculae (Figure 1b(ii)). The trabecular bone of the central diploe is an energy absorbing lightweight meshed structure that provides cushioning, shear strength, and separation between the cortical plates in order to increase the inertial characteristics (bending strength) that allows the three-layered structure to endure mainly bending loads. Moreover, such a structure that makes the trabecular bone a highly porous, heterogeneous, and anisotropic material to absorb the external shock contains bone marrow and skull vasculatures. The density of cortical and trabecular bones range between 1.8 and 2.2 and 0.3 and 1.3 g/cm^3^, respectively [48]. Since the density of the cortical bone is higher than that of the trabecular bone, energy is mostly absorbed by the cortical bone, whereas the porosity is higher in the trabecular bone and, therefore, scattering occurs within the trabecular diploe layers [49,50].

The mechanical and acoustic characteristics of the skull bone described in the literature can be summarized as follows: the characterization of the human skull in terms of the speed of sound and thickness were explored in several studies [25,51,52,53,54]; the longitudinal speed of sound and the acoustical attenuation coefficient of human calvaria were studied at frequencies ranging from 0.27 to 2.526 MHz [29]; the speed of sound in cortical bone is within the range of 2880–4220 m/s [55,56]; for the trabecular bones, the speed of sound is lower (2000–3000 m/s) and the attenuation is higher (15–30 dB/MHz/cm) as compared to those of the cortical bone [57]; in [58], the insertion loss and the elastic constants of the skull were measured; studies have also been performed to attain the optical properties of the skull bones [58,59,60,61,62]; the optical properties of human cranial bone were measured using the integrating sphere technique in [60]; the reduced scattering coefficient of the human skull follows *μ*_s_’(*λ*) = 1533.02 × *λ*^−0.65^ in the wavelength range, *λ* = 800–1000 nm [63]; in the near infrared region (600–900 nm), the reduced scattering and absorption coefficient of the human skull are in the range of 0.2–1.2 cm^−1^ and 20–25 cm^−1^, respectively; the predominant ultrasound attenuation mechanism in the trabecular bone is scattering, while the absorption is considered to be a major attenuation mechanism in the cortical bone; the cortical bones exhibit higher optical scattering and absorption as compared to the trabecular bones [64]; a generalized pattern of acoustic transmittance and optical intensity decay as a function of time are shown in [64]—in this article, it is shown that due to a more prominent effect of optical scattering, the intensity decay has a slower rate in trabecular bones compared to acoustic transmittance. Although several studies have explored different skull properties—e.g., the geometry, scattering coefficient, speed of sound, insertion loss, and transmission dispersion [58,65,66]—the effect of the skull in the illumination path and the acoustic detection path, separately, has not been investigated quantitatively in a photoacoustic transcranial imaging experiment.

In this study, we investigate the feasibility of transcranial photoacoustic imaging by studying the effect of the skull in both the illumination path and the acoustic detection path, and determine the maximum skull thickness through which the accurate photoacoustic imaging of the structure and vasculature is feasible. Our investigation aims to explore and quantify the deterioration of PA images owing to the obstacle of the skull bone in three paths: (i) light illumination, (ii) acoustic propagation, and (iii) both light illumination and acoustic propagation.

## 2. Materials and Methods

### 2.1. PAI System

The PAI system used in this study composes of Phocus MOBILE, a 10 Hz Nd:YAG tunable laser (OPOTEK, Carlsbad, CA, USA) in the range 690 to 900 nm, that is controlled by an internal optical parametric oscillator (OPO). A silica fiber bundle consisting of 100 fibers with a total diameter of 1 cm has been used for light delivery. The average output energy at the fiber end was measured as ~20 mJ using an energy meter (QE12SP-H-MT-D0, Gentec-EO, Quebec, QC, Canada). The spot size was 8 mm on the skull piece, and the spot size on the target could not be measured since it was embedded in the phantom. Considering the numerical aperture of the optical fiber, the divergence of the light was ~30°.

Since the acoustic window near the temporal or occipital region is with a diameter of ~3 cm [67], phased array transducers are preferred. A phased array sensing surface has a smaller footprint area as compared to linear and curvilinear arrays. Moreover, a phased array provides a wider field of view and it has dynamic focusing capabilities, which increase the flexibility of scanning without or with a minimal mechanical movement of the array. We used a 64-element phased array P4-2 transducer probe (Philips Healthcare, Ville Platte, LA, USA) with a 2.5 MHz center frequency. The transducer was held in water inside an open top box using clamps. The clamps were attached to a two-axis mechanical stage for scanning. The probe was scanned in the *y*-axis to cover a distance of 2 cm with 48 total steps and a step size of 0.4 mm. The Vantage 128 imaging platform (Verasonics Inc., Kirkland, WA, USA) was used for the data acquisition and image processing.

### 2.2. Skull Tissue Preparation

There are structural differences between the sheep skull and human skull in terms of thickness, content, and architecture. Despite the differences, the structural components (diploe, outer, and inner table) in human and sheep skulls are similar. Here, we evaluate the effect of skull thickness on the reconstructed PA image; therefore, maintaining the skull thickness is important. To achieve similar thicknesses of the human skull at different ages, we have chosen the frontal skull bone of sheep head and mechanically configured the sheep skull to be flat and representative of human skull thicknesses. Three different skull thicknesses of 0.7, 1.0, and 1.3 mm were used. The skull samples were collected from ex vivo sheep heads. Using a Hole Dozer general purpose circular saw (Milwaukee Electric Tool, Brookfield, WI, USA), skull pieces with a diameter of 5 cm were cut. Later, 1.5 cm areas on the skull pieces were thinned down to the desired thickness using a drill bit. The thickness of each skull sample after preparation was measured using a H-2780 digital screw gauge (ULINE, Milton, ON, Canada) at 5 different points and averaged.

### 2.3. Phantom Experiments

**Figure 2:**
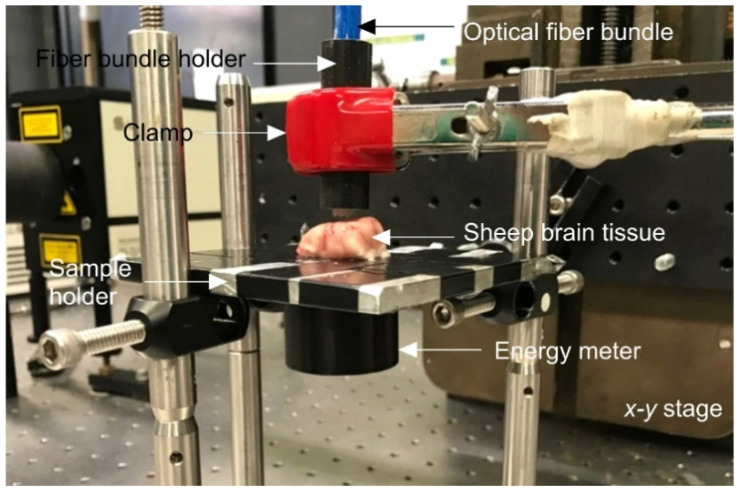
Experimental setup of the optical transmittance characterization to find the thickness of the tissue-mimicking phantom that is optically equivalent to brain tissue.

We embedded an imaging target in a brain tissue-like mimicking phantom with optical properties similar to those of the brain tissue. To determine the light attenuating characteristics of the brain tissue, slices of sheep brain with a thicknesses between 0.5 and 1.75 cm were prepared. The experimental setup is shown in Figure 2. The ex vivo brain tissue was held on a metal plate with a circular hole. An energy meter (QE8SP-B-BL-INT-D0, Gentec-EO Inc., Quebec City, QC, Canada) was coaxially aligned to the optical fiber bundle through the hole; the surface of the sensor was protected with an optically transparent thin film. The brain tissue-mimicking phantom was realized by mixing gelatin (to represent the acoustic properties) and sugar-free psyllium hydrophilic mucilloid fiber (Metamucil, P&G, Cincinnati, OH, USA) (to represent the tissue optical attenuation and echogenicity). First, 8% gelatin was dissolved in water, followed by 4% fiber in a transparent one-side-open cubic acrylic box (Lanscoery, Monterey Park, CA, USA) [68]. Tissue-mimicking phantoms with thicknesses of 1, 2, 3, 4, and 5 cm were prepared.

**Figure 3:**
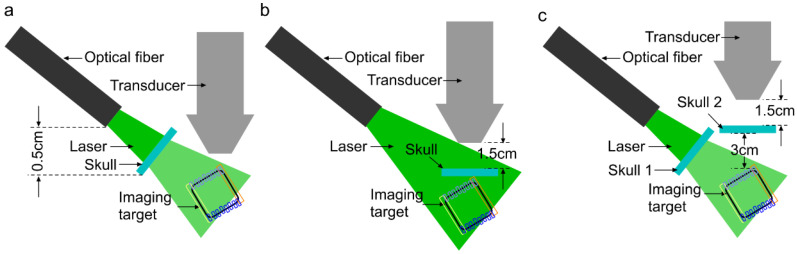
Schematic setups for imaging the square loop phantom with the skull as a barrier in: (**a**) the optical path, (**b**) the acoustic detection path, and (**c**) both the optical and acoustic detection paths. The optimized distances are calculated in Section 3.

**Figure 4:**
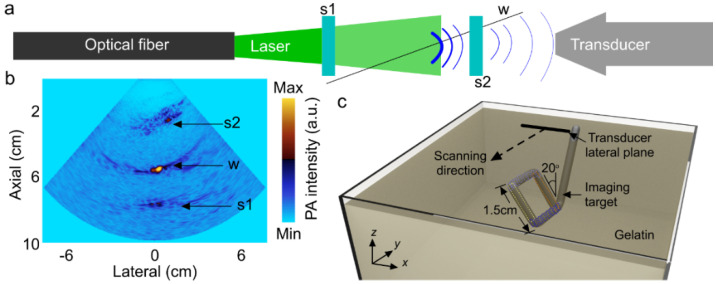
Experimental setup to find the optimum distance between the transducer and skull piece 2, and between the optical fiber and skull piece 1. (**a**) Schematic of the experimental setup, (**b**) photoacoustic (PA) image of the wire phantom in water with the optimum position of the transducer and optical fiber, and (**c**) a 3D model of the square loop imaging target in gelatin. Imaging target is slanted at 20° from the *z* axis. Scanning direction is along the *x* axis. Transducer lateral plane is along the *y* axis. s1: skull piece 1; s2: skull piece 2; w: polyactic wire. The distance between the transducer and s2 is <1.5 cm, the distance between the transducer and the imaging target is <3 cm, and the distance between the optical fiber bundle and s1 is 0.5 cm.

Next, we evaluated the effect of skull as a dispersive barrier in three paths: (i) light illumination, (ii) acoustic propagation, and (iii) both light illumination and acoustic propagation (see Figure 3a–c). The imaging target was a square loop of a 0.5 mm-thick copper rod covered with an insulating dark jacket, polyactic material (see Figure 4c), and held in the tissue-mimicking phantom mixture at a 20° angle versus the probe viewing plane at a 1 cm distance from the surface of the phantom. We initially tried to implement a 3D structure of blood vessels embedded within the gelatin phantom. Since the blood vessels were positioned in a three-dimensional coordinate, we could not inject blood evenly inside the blood vessels. This made the evaluation of the results difficult. We then used a thin plastic tube to represent the blood vessels. In addition to the fact that these tubes had additional absorption, the stationary blood within the tubes started forming sediment at the bottom of the tube. Therefore, we utilized a solid imaging target instead of the actual blood vasculature. According to the literature, the absorbance of polyactic material (with a normalized absorbance ~25% [69]) at the imaging wavelength of 690 nm is close to the absorbance of blood (with the normalized absorbance of ~20% [70]).

The ultrasound probe was horizontally scanned across the imaging target by manually rotating the *x*-axis knob of the *x*-*y* stage with a step size of 1 mm. Each 3D image was comprised of 50 2D B-scan images, which were later compiled into a 3D volume in Slicer 4.10 [71]. In the 3D slicer, the projection of the 3D volume was automatically adjusted to the default intensity range and, therefore, the intensity map had to be corrected. The process of correcting the intensity projection is as follows. Initially, we selected a B-scan frame for each configuration, where a specific portion of the square loop can be visualized without any post-processing; in this case, we have chosen the B-scan frames that correspond to the light green and orange dotted region of interests (ROIs), as shown in Figure 3. We then calculated the average intensity value of those specific frames and, later, applied them as a gain (intensity modifier) to the corresponding 3D volumes to project the corrected intensity map.

### 2.4. Quantitative Evaluation Parameters

We evaluated the PA images quantitatively in terms of the average intensity attenuation (AIA), smoothness (S), and image distortion (ID). These parameters were evaluated for the entire region on the square loop imaging phantom. AIA is defined as the averaged intensity in the specified area; S (can be viewed as lack of roughness) is extracted from the line profile across the specified ROI with and without the skull PA images, and is defined as the correlation between the peak values of the corresponding normalized line profiles. ID is defined as sum of the square of the regression (SSR) of the rising and falling edges of the line profile in the US image. Additionally, the image distortion in the PA images was defined as the difference between the contour profile of the PA image with and without the presence of the skull.

The processing protocol was as follows. The imaging target orientation was such that the light blue ROIs (indicated in both Figure 3 and Figure 4c) were closest to the surface of the US probe (~4.5 cm) and the dark blue ROIs were the furthest away (~6 cm). We initially extracted several line profiles within the light green and orange ROIs (indicated in Figure 3 and Figure 4c) and averaged them. The averaged line profile represents the signal intensity decay as a function of the distance between the imaging target and the transducer probe (i.e., 4.5 to 6 cm). Next, we extracted several parallel line profiles within each of the light and dark blue ROIs. The average and standard deviations of the extracted values 4.5, 5, 5.5, and 6 cm were presented in tables and figures. It is of note that the transducer probe was scanned in one direction. As a result, the light green and orange ROIs are located along the lateral plane of the transducer surface, and hence the entire ROI can be visualized in a 2D image, whereas the light and dark blue ROIs are seen as moving dots (due to the location of the ROIs in the cross-sectional imaging plane). Therefore, the light green and orange ROIs are represented by large rectangular boxes, whereas the light and dark blue ROIs are represented by small rectangular boxes.

## 3. Results and Discussion

Initially, to find out the optimum distance between the transducer and skull, we imaged a copper wire coated with polyactic jacket as the imaging target (see Figure 4a). By finding the optimum distances, any reflecting artifact overlapping with the signal coming from the imaging target were avoided. The phantom was held inside a transparent plastic container and fixed to the optical table. The transducer probe and skull pieces were held using optical rods and fixed to a customized *x-y* stage, made in the machine shop at Wayne State University. The experiment was performed in two stages: (1) First, the position of the sample was fixed with respect to the wire phantom and the transducer probe (5 cm away from the phantom) was moved towards the skull piece 2, using a mechanical stage in the *y*-axis with steps of 5 mm; this configuration was used to optimize the distance between the transducer and skull piece 2. (2) Once we determined the optimum position of the transducer with respect to skull piece 2, both the transducer and skull piece 2 were moved simultaneously from a distance of 5 cm towards the wire phantom, while the distance between the transducer and skull piece 1 was fixed; this configuration provided information regarding the US signal behavior generated from the phantom as a function of depth while the transducer was at a constant distance from skull piece 1. The optimum configuration was as follows: the P4-2 probe was at least 1.5 and 3 cm away from skull piece 2 and the imaging target, respectively; the optical fiber bundle was 0.5 cm away from skull piece 1 (see Figure 3a).

**Figure 5:**
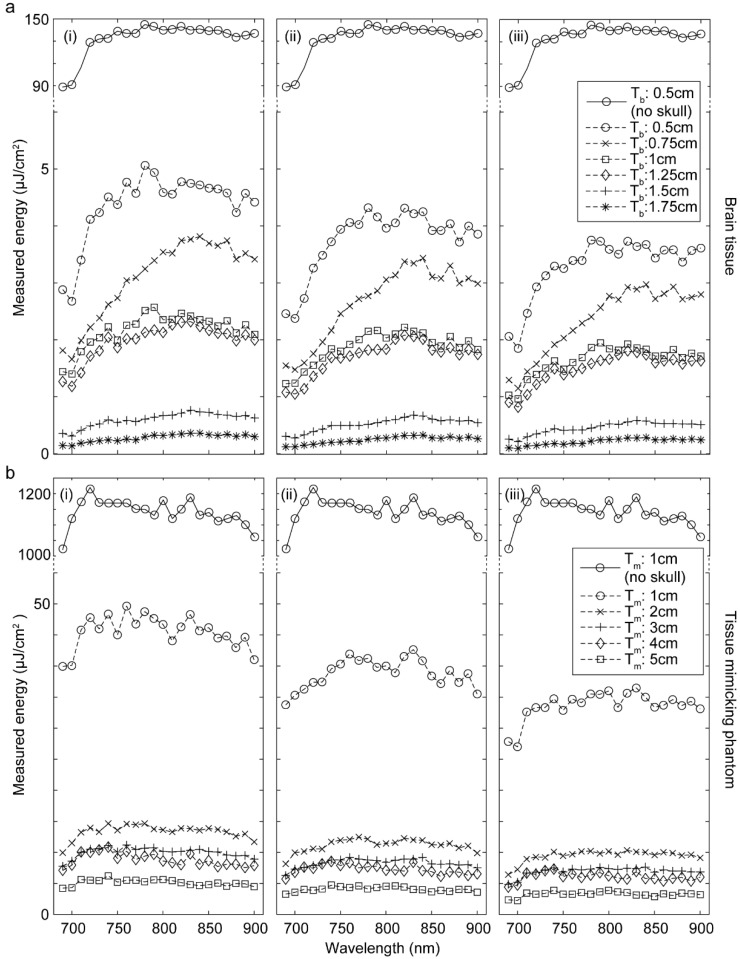
Optical energy measured with different thicknesses of (**a**) sheep brain tissue and (**b**) the brain tissue-mimicking phantom (gelatin + fiber) with a skull bone to block the illumination path with a thickness of (**i**) 0.7, (**ii**) 1.0, or (**iii**) 1.3 mm. Optical fiber bundle was placed on top of the brain tissue or the brain-like tissue-mimicking phantom. T_b_: brain tissue thickness; T_m_: tissue-mimicking phantom thickness.

Next, using the experimental setup shown in Figure 2, we determined the thickness of the tissue-mimicking phantom that optically models the brain tissue. In this setup, the optical fiber bundle was placed right on top of the brain tissue or the brain-like tissue-mimicking phantom. With the laser energy measured (at the distal end of the fiber it was 30 mJ), we were able to measure the optical attenuation of different thicknesses of the brain tissue (i.e., T_b_: 0.5 cm to 1.75 cm) and the brain tissue-mimicking phantom (i.e., T_m_: 1 cm to 5 cm) through skull samples with thicknesses of 0.7, 1.0, and 1.3 mm; any thicknesses beyond the above thicknesses entirely blocked the light and thus were not considered in our experiments. The results shown in Figure 5 indicate that a 5 cm-thick tissue-mimicking phantom optically resembles the ~5 mm brain tissue.

**Figure 6:**
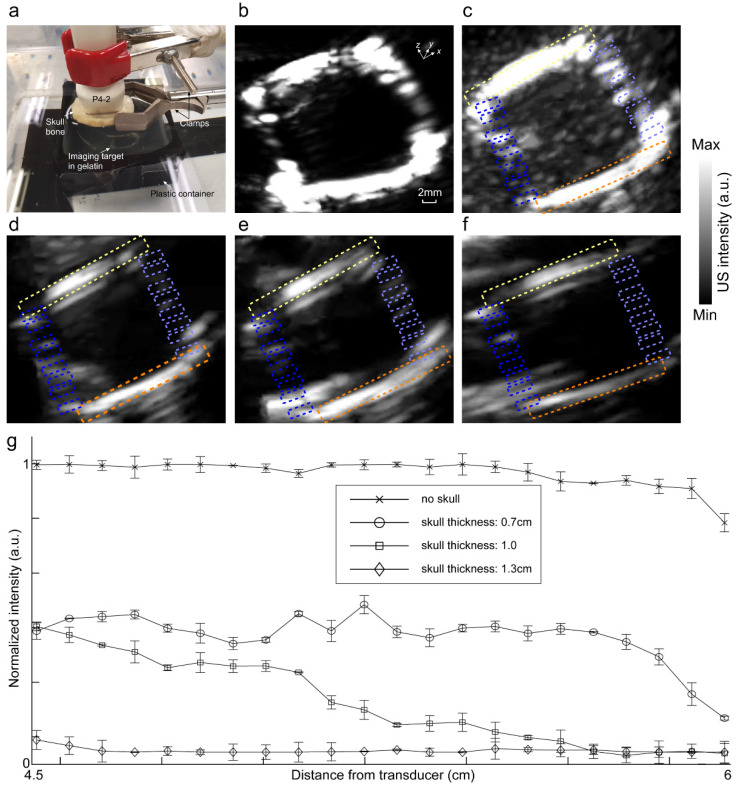
Image intensity attenuation and distortion analysis in a transcranial ultrasound imaging experiment. (**a**) Experimental setup. US image of the square imaging target (**b**) in gelatin and (**c**) in gelatin with the fiber mixture. Ultrasound images of the square imaging target in the gelatin with the fiber mixture through a skull piece with a thickness of (**d**) 0.7, (**e**) 1.0, and (**f**) 1.3 mm. (**g**) Average line profiles within the ROIs indicated with (i) light green, (ii) dark blue, (iii) light blue, and (iv) orange dotted boxes depicted in (**c**–**f**). Light green and orange ROIs are at the same depth from the transducer. This distance increases from the light to dark blue ROIs from 4.5 to 6 cm. The transducer was at the distance of 1.5 cm from the skull, and the skull was at the distance of 3 cm from the imaging target. ROI: region of interest.

We then evaluated the ultrasound images of the square loop phantom when the transducer was held 1.5 and 3 cm away from the skull piece and the square loop phantom, respectively; when the optical fiber was placed 0.5 cm away from the skull piece (see Figure 3); and when the orientation of the phantom was at an angle of 20° (Figure 4c) to the viewing plane of the probe. We imaged the target in both gelatin (see Figure 6b) and gelatin with fiber mixture (see Figure 6c). The US images without the skull clearly present the morphology of the square loop. In Figure 6d–f, a skull piece with thicknesses of 0.7, 1.0, or 1.3 mm were used when the square loop target was in gelatin with the fiber mixture. The ROIs on the squared loop phantom image were chosen to evaluate the effect of the skull aberration. The attenuating and aberrating effects of the skull are shown in Figure 6g. A summary of the quantitative evaluation is provided in Table 1.

**Figure 7:**
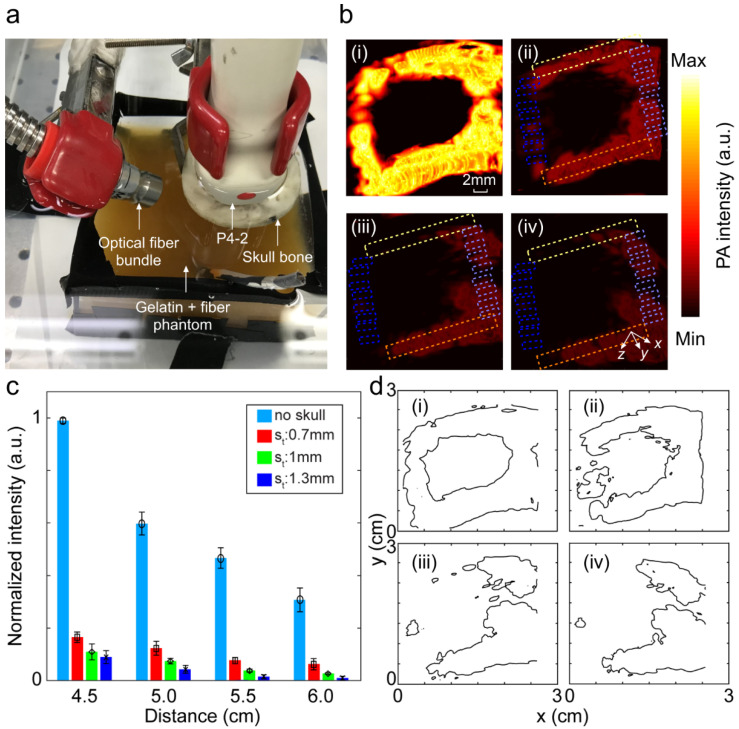
Image distortion analysis in photoacoustic imaging when the skull blocks the acoustic detection path. (**a**) Experimental setup. (**b**) PA image of the square loop imaging target (i) without skull, (ii) with a 0.7 mm skull, (iii) with a 1.0 mm skull, and (iv) with a 1.3 mm skull. (**c**) Average PA signal intensity within the ROIs depicted in (**b**i–**b**iv) as a function of the distance between the transducer surface and the imaging target (4.5, 5, 5.5, and 6 cm). Light green and orange ROIs are at the same depth from the transducer. This distance increases from the light to dark blue ROIs from 4.5 to 6 cm. The transducer was at a distance of 1.5 cm from the skull, and the skull was at a distance of 3 cm from the square target. (**d**) Contour map of the PA images representing the skull-induced deformation (i) without the skull, (ii) with a 0.7 mm skull, (iii) with a 1.0 mm skull, and (iv) with a 1.3 mm skull. The transducer was at a distance of 1.5 cm from the skull, and the skull was at a distance of 3 cm from the square loop imaging target. S_t_: skull thickness.

Next, we studied the distorting effects of the skull on the PA images when it blocked only the acoustic detection path. The experimental setup as well as the PA images of the square loop imaging target with different thicknesses of the skull are shown in Figure 7a,b, respectively, and a summary of the quantitative evaluation is provided in Table 2. A bar chart to show the attenuating effect of skull as a function of the distance between the transducer and the imaging target for different skull thicknesses is provided in Figure 7c. Here, thicker skulls (1 and 1.3 mm skulls) impacted the PA signal intensity to decay abruptly at higher depths and, therefore, the average intensity decays faster along the light green and orange ROIs towards the dark blue ROIs. The contour profiles of the PA images to present the severity of the structural deformation with different thicknesses of the skull is provided in Figure 7d.

**Figure 8:**
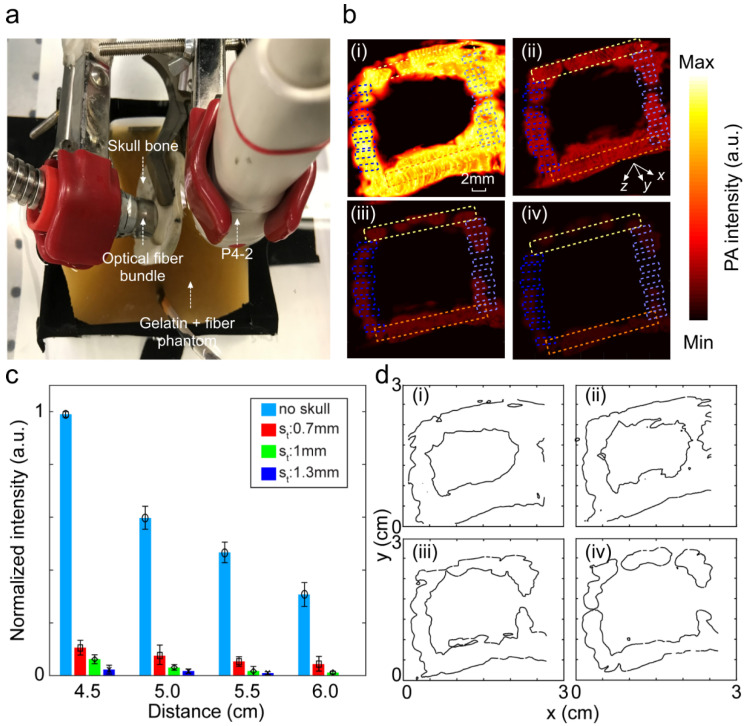
Image distortion analysis in photoacoustic imaging when the skull blocks the light illumination path. (**a**) Experimental setup. (**b**) PA image of the square loop imaging target (i) without the skull, (ii) with a 0.7 mm skull, (iii) with a 1.0 mm skull, and (iv) with a 1.3 mm skull. (**c**) Bar chart of the average PA signal intensity within the ROIs is depicted in (**b**i–**b**iv) as a function of the distance between the transducer surface and imaging target (4.5, 5, 5.5, and 6 cm). (**d**) Contour map of PA images representing the skull-induced deformation (i) without the skull, (ii) with a 0.7 mm skull, (iii) with a 1.0 mm skull, and (iv) with a 1.3 mm skull. Light green and orange ROIs are at the same depth from the transducer. This distance increases from the light to dark blue ROIs from 4.5 to 6 cm. S_t_: skull thickness.

We then studied the distorting effects of the skull on the PA images when it blocked only the light illumination path. The experimental setup as well as the PA images of the square loop imaging target with different thicknesses of the skull are shown in Figure 8a,b, respectively, and a summary of the quantitative evaluation is provided in Table 3. A bar chart to show the attenuating effect of the skull as a function of the distance between the transducer and the imaging target for different skull thicknesses is provided in Figure 8c. Unlike the effect of the skull on the acoustic path, here the PA average intensity attenuation is higher; however, the intensity decay rate as a function of depth is comparatively lower. The contour profiles of the PA images to present the severity of structural deformation at different thicknesses of the skull is provided in Figure 8d.

**Figure 9:**
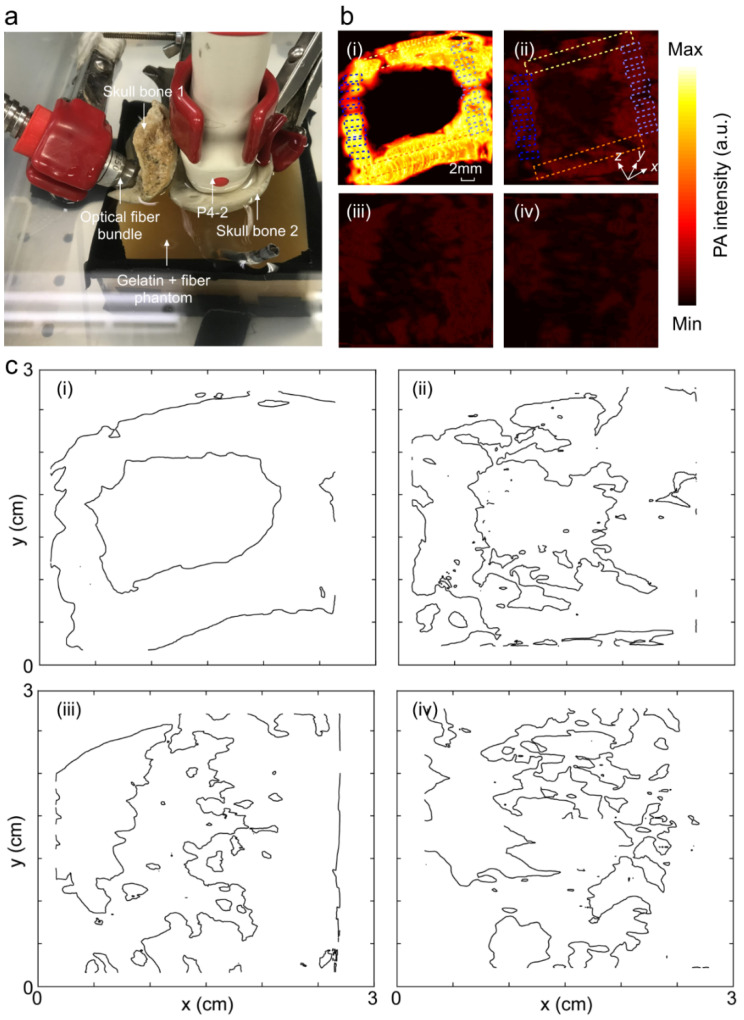
Image distortion analysis in photoacoustic imaging when the skull blocks both the light illumination and acoustic detection paths. (**a**) Experimental setup. (**b**) PA image of the square loop imaging target (i) without the skull, (ii) with a 0.7 mm skull, (iii) with a 1.0 mm skull, and (iv) with a 1.3 mm skull. (**c**) Contour map of the PA images representing the skull-induced deformation (i) without the skull, (ii) with a 0.7 mm skull, (iii) with a 1.0 mm skull, and (iv) with a 1.3 mm skull. The transducer was at a distance of 1.5 cm from the skull and the skull was at a distance of 3 cm from the square loop imaging target. Light green and orange ROIs are at the same depth from the transducer. This distance increases from the light to dark blue ROIs from 4.5 to 6 cm.

Finally, we studied the distorting effects of the skull on the PA images when it blocked both the acoustic propagation and the light illumination paths. The experimental setup as well as the PA images of the square loop imaging target with different thicknesses of the skull are shown in Figure 9a,b, respectively, and the summary of the quantitative evaluation is provided in Table 4.

The findings of this experiment were as follows: (i) The light was completely diffused inside the tissue-mimicking phantom after passing through the skull pieces, therefore a homogenous illumination of the target phantom was obtained. (ii) The horizontal sides of the phantom generated a higher PA signal amplitude compared to the vertical sides because of the transducer viewing plane. (iii) The only skull tissue that allowed seeing the structure of the imaging target accurately was the skull piece with a 0.7 mm thickness; with the 1.0 mm skull, the shape of the imaging target was almost visible (Figure 9c(iii)), and with the 1.3 mm skull (Figure 9c(iv)), the structure of the square loop target in the image was totally distorted and attenuated to such an extent that the target was not comprehensible.

**Figure 10:**
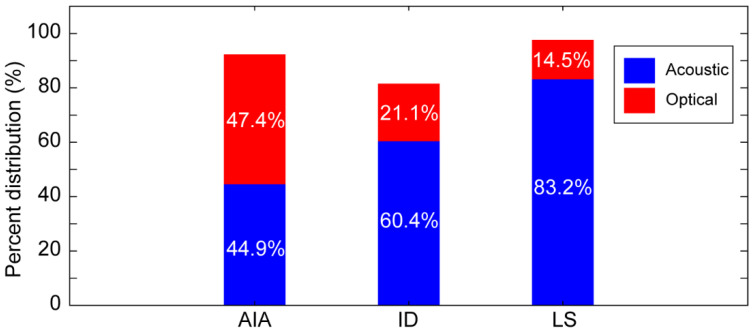
Percent distribution of the evaluating parameters from the acoustic and optical paths when both paths are blocked by the skull (thickness: 0.7 mm). AIA: average intensity attenuation; ID: image distortion; LS: lack of smoothness (1-S).

The goal of this study was to evaluate the combined effect of the skull layers on the acoustic and optical attenuation. We used the architecture of the skull and its layer information, published in research articles, to explain the results. Furthermore, creating a ~1 cm-diameter flat cortical and trabecular layer tissues, thinned down to a millimeter thickness, requires sophisticated machinery, especially with the brittle nature of the skull layers, which was not available to us. A quantitative evaluation of the percent distribution of the acoustic and optical path blocked towards the overall evaluation parameters presented in Table 4 is shown in Figure 10. The individual contribution has been calculated based on their respective values presented in Table 2 and Table 3.

The quantitative evaluations in Figure 7, Figure 8, and Figure 10 show that blocking the acoustic propagation affects the PA signal more significantly as compared to blocking the illumination path. According to the literature, the optical illumination path is mainly affected by the cortical layers in the form of optical energy attenuation due to the higher absorption, scattering, and reflection of this layer [63,72,73,74,75], whereas the trabecular structure of diploe layer induces more acoustic scattering and insertion loss. Moreover, among the three quantitative measures, image distortion and the lack of smoothness are the major consequences of the diploe layer that constitutes the majority thickness of thick bones, whereas image average intensity attenuation is the consequence of the cortical layer [51,52,76,77,78,79]. In other words, blocking the optical path more significantly contributes to the amplitude decay, while blocking the acoustic propagation path contributes mainly to the distortion of the morphological map of the imaging target [29,52,80,81]. The combined significant acoustic distortion in the diploe layer and the optical attenuation in the cortical–diploe or cortical–tissue interfaces make the use of photoacoustic transcranial imaging challenging in animals with thicker skulls or in humans.

## 4. Conclusions

While photoacoustic imaging has shown great promise in the transcranial brain imaging of small animals, it is still underdeveloped for clinical us due to the presence of the skull. We studied the distorting effect of the skull when it is in the optical illumination path, acoustic detection path, and both simultaneously. We determined the maximum thickness of the skull through which PAI is feasible, such that the structure of the imaging target with no post processing is distinguishable; this thickness was ~0.7 mm. Utilizing sophisticated reconstruction algorithms as well as signal/image enhancement techniques [42,82,83,84,85,86], imaging through thicker skull tissues will be possible. Due to the complexity of creating flat cortical and trabecular layer tissues, we studied the combined effect of the skull layers on the acoustic distortion and optical attenuation. We concluded that the average intensity attenuation and distorting effect of the skull due to the blockage of the acoustic path is ~2.5% less and ~39.3% greater than those of the illumination path, respectively. These results can help in designing a more efficient photoacoustic imaging system suitable for transcranial brain imaging.

## Figures and Tables

**Table 1:** Summary of the quantitative evaluation of the US imaging through the skull in gelatin with a fiber mixture with different thicknesses. Please see the definition of the quantitative parameters in Section 2.4.

Skull Thickness (mm)	Image Average Intensity Attenuation (%)	Image Distortion (%)	Smoothness (%)
0.7	46.5 ± 1.30	32.5	50 ± 1.47
1.0	48.23 ± 3.74	35.6	10.7 ± 1.26
1.3	78.4 ± 4.23	56.28	1.8 ± 0.89

**Table 2:** Summary of the quantitative evaluation of PA imaging through the skull in gelatin with the fiber mixture when the acoustic propagation path is blocked with skull pieces with different thicknesses.

Skull Thickness (mm)	Image Average Intensity Attenuation (%)	Image Distortion (%)	Smoothness (%)
0.7	88.42 ± 1.12	32.12	5.74 ± 2.71
1	92.59 ± 0.27	72.45	4.41 ± 2.05
1.3	95.17 ± 0.21	79.73	2.46 ± 1.87

**Table 3:** Summary of the quantitative evaluation of the PA imaging through the skull in gelatin with the fiber mixture when the light illumination path is blocked with different thicknesses of skull.

Skull Thickness (mm)	Image Average Intensity Attenuation (%)	Image Distortion (%)	Smoothness (%)
0.7	91.10 ± 2.98	11.26	32.5 ± 1.56
1	95.07 ± 2.24	18.67	6.18 ± 1.06
1.3	97.03 ± 1.89	23.91	2.46 ± 0.74

**Table 4:** Summary of the quantitative evaluation of the PA imaging through the skull in gelatin with the fiber mixture when both the light illumination and acoustic propagation paths are blocked by the skull.

Skull Thickness (mm)	Image Average Intensity Attenuation (%)	Image Distortion (%)	Smoothness (%)
0.7	92.3 ± 2.83	81.6	1.76 ± 1.38
